# Leadership experiences and practices of South African health managers: what is the influence of gender? -a qualitative, exploratory study

**DOI:** 10.1186/s12939-018-0859-0

**Published:** 2018-09-18

**Authors:** Maylene Shung-King, Lucy Gilson, Chinyere Mbachu, Sassy Molyneux, Kelly W. Muraya, Nkoli Uguru, Veloshnee Govender

**Affiliations:** 1 Health Policy and Systems Division, School of Public Health and Family Medicine, University of Cape Town, Cape Town, South Africa; 2 Department of Global Health and Development, London School of Hygiene and Tropical Medicine, London, UK; 3 College of Medicine, University of Nigeria Enugu Campus, Enugu, Nigeria; 4 KEMRI-Wellcome Trust Research Programme, Kilifi, Kenya; 5 Alliance for Health Policy and Systems, World Health Organization, Geneva, Switzerland

**Keywords:** Gender and leadership in health, Gender, race and professional hierarchy and health leadership, South African health leaders and gender, Intersectional social identities and health leadership

## Abstract

**Background:**

The importance of strong and transformative leadership is recognised as essential to the building of resilient and responsive health systems. In this regard, Sustainable Development Goals (SDG) 5 prioritises a current gap, by calling for women’s full and effective participation and equal opportunities for leadership, including in the health system. In South Africa, pre-democracy repressive race-based policies, coupled with strong patriarchy, led to women and especially black women, being ‘left behind’ in terms of career development and progression into senior health leadership positions.

**Methods:**

Given limited prior inquiry into this subject, we conducted a qualitative exploratory study employing case study design, with the individual managers as the cases, to examine the influence of gender on career progression and leadership perceptions and experiences of senior managers in South Africa in five geographical districts, located in two provinces. We explored this through in-depth interviews, including life histories, career pathway mapping and critical incident analysis. The study sample selection was purposive and included 14 female and 5 male senior-managers in district and provincial health departments.

**Results:**

Our findings suggest that women considerably lag behind their male counterparts in advancing into management- and senior positions. We also found that race strongly intersected with gender in the lived experiences and career pathways of black female managers and in part for some black male managers. Professional hierarchy further compounded the influence of gender and race for black women managers, as doctors, who were frequently male, advanced more rapidly into management and senior management positions, than their female counterparts. Although not widespread, other minority groups, such as male managers in predominantly female departments, also experienced prejudice and marginalisation.

Affirmative employment policies, introduced in the new democratic dispensation, addressed this discriminatory legacy and contributed to a number of women being the ‘first’ to occupy senior management positions. In one of the provinces, these pioneering female managers assumed role-modelling and mentoring roles and built strong networks of support for emerging managers. This was aided by an enabling, value-based, organisational culture.

**Conclusion:**

This study has implications for institutionalising personal and organisational development that recognise and appropriately advances women managers, paying attention to the intersections of gender, race and professional hierarchy. It is important in the context of national and global goals, in particular SDG 5, that women and in particular black women, are prioritised for training and capacity development and ensuring that transformative health system policies and practices recognise and adapt, supporting the multiple social and work roles that managers, in particular women, play.

## Background

Leadership is widely regarded as an important facet in health system strengthening and is a critical element in building responsive and resilient health systems [24, 60]. Whilst management and leadership are often referred to as ‘two sides of the same coin’, in this paper we embrace the notion of leadership as a distinct phenomenon and an essential part of the portfolio of competencies of managers, including health managers [15, 23, 48]. Whilst we recognise the phenomenon of distributed leadership, meaning that leadership resides in multiple levels of the health system and not just in those who hold formal management positions [4], this paper focuses on the perspective and experiences of formally designated managers.

Leadership literature speaks to different forms of leadership and leaders with different characteristics and styles [10, 30, 48]. Beyond individuals, the importance of teams as collective units of leadership is also well-recognised in contemporary leadership literature [2, 42, 57]. Health systems, meanwhile, are recognized as complex adaptive systems, encompassing interdependent organisations and institutions which are diverse in form and structure [1, 34]. These complex adaptive systems shape and are shaped by the behaviours of individuals and teams located within them [3, 27, 43, 49, 50]. Given the integral position of the health sector in broader society, this interdependence also extends more widely, with the health system and all who work in it influencing and being influenced by wider societal factors.

As leadership essentially resides in people, social identities are among the multiple influences that impact on leadership behaviours and experiences within the health system [15, 33]. The gendered nature of health systems [59], with different health professions strongly dominated by either males or females, is one such influence over the shape and form of leadership. Yet gender is largely ignored in the general and health-specific management literature, despite global calls for gender-parity in organisational leaders [55]. There is also little research to understand the influence of gender on health system leadership.

This paper, then, explores the extent to which gender influences the perceptions and experiences of health managers, through a qualitative, exploratory study undertaken in South Africa. As human resource development in the South African health sector is deeply rooted in a race-based, gendered and professionally hierarchical political and economic history [12], this is a particularly interesting context in which to conduct this work.

The *Apartheid* (i.e. separateness) government in South Africa, characterised by repressive race-based policies favouring a White minority grouping, was replaced in 1994 by a constitutional democracy [21]. Prior to democracy, *apartheid* resulted in a civil service, including the health sector, where Whites, and predominantly males, held the majority of posts, including the most senior and powerful positions [12]. At a professional level, the South African health system had a bias towards doctors as the de facto leaders of the health care team [12]. This triumvirate of social identities resulted in White male doctors occupying the majority of high level health management posts in hospitals and in national, provincial and local authority Departments of Health. This bias also extended to support-service positions such as human resources, finance and supply chain, where management positions were mostly held by White, often Afrikaans speaking (the dominant language of the Apartheid government), males. In general, the numbers of women- and black doctors lagged behind. For black women in particular, nursing and teaching were often the only professional career options available [47]. Post-democracy in 1994, there was large-scale cross-sectoral transformation, including the development of policies to redress race and gender-linked workplace imbalances and actively affirm the appointment of Blacks, women and those with disabilities. This resulted in important changes in the public-sector health management profile [16].^1^ It was against this backdrop of systematic race-based and gendered human resource development in the health sector, that we examined the role of gender in leadership. Our fieldwork was conducted in 2016, just over 20 years into democracy.

## Methods

### Research design

We employed a qualitative case study approach [13], which is appropriate for studies exploring complex social phenomenon (e.g. leadership, gender, social norms etc.), allowing for description and interpretation of participant experiences in the natural setting and context in which they occurred. A defining characteristic and advantage of a case study approach is a focus on depth to obtain a rich complete picture, often requiring a variety of data tools. In this study, health managers were the cases and thus the ‘units of analysis’. Their experiences of gender, in relation to their career pathways and leadership experiences and practices, were explored in-depth in their district and provincial contexts and allowed for conclusions to be drawn about these health managers in their specific contexts. This was appropriate given the limited research conducted on understanding gender and leadership in low- and middle-income countries (LMICs) and the purpose of the study was exploratory, to provide initial, descriptive data. This could be the basis for the generation of new hypotheses and research questions for further research. A key limitation of case study design and applicable to the present study is that, given the emphasis on in-depth content, the research cannot be feasibly conducted on a large scale and is not necessarily generalisable to other contexts.

### Study setting, sampling and selection of research participants

Nineteen senior health managers (our cases) were interviewed from two provincial departments of health. We were interested in leaders who routinely translate strategic plans that are set at higher levels (national or provincial) into operational plans for implementation at their own level and below. Therefore, we focused on understanding the experience and practice of relatively senior health system leaders in decentralised governance and administrative structures (i.e. at provincial and district level).

Selection of participants was purposive [25]. Purposive sampling is often used in qualitative research to identify a particular group of people who possess certain characteristics or are in circumstances pertinent to the phenomenon being studied [25]. The sampling frame comprised provinces and districts (five districts - two metropolitan and three rural - across two provinces). We selected an urban district to sample from in each province and included a selection of participants from three rural districts in one of the provinces. These different urban and rural settings provided a range and depth of experiences of relevance to our phenomena of interest. Participants were selected to reflect diversity in gender, race, age and professional background. A listing of senior managers was obtained from the provincial and district health authorities and suitable participants were invited to participate in the study.

The two provinces in which our study was conducted were similar in economic status, but with different party-political histories. The two provinces have had a very different leadership trajectory over the past 25 years; one had a stable core of senior leadership and the other a frequently changing senior leadership.

Within districts, selection of cases (i.e. senior health managers) was guided by the Galer and Vriesendorp typology [23], which identifies four levels of health system leadership, and locates middle and senior managers in the second and third levels, respectively. In this typology, senior managers^2^ are characterized as ‘organizational decision-makers’, for whom strategic thinking, coaching others, managing external consultants contracted to do work, managing conflict and using reflective skills are critical leadership skills. In South Africa, while senior managers in the district are responsible for overseeing the delivery of health services, senior managers in the provincial structures have supervisory roles, and provide guidance and frameworks to support district operations.

### Conceptual guidance

We drew on selected conceptual ideas and frameworks to guide the different aspects of our interviews (see below) and our analysis.

Gender is understood as ‘… the socially constructed roles, behaviours, activities and attributes that a given society considers appropriate for males, females and other genders—affects how people live, work and relate to each other at all levels, including in relation to the health system’ ([44], p. 1). This understanding of gender has several important implications for how it is examined, including in this paper. Firstly, gender is relational and is shaped by access to and distribution of resources and power between men and women. Second, gender norms and roles are dependent on context and vary across time. Third, differences in gender relations are also shaped by intersections of gender with other social constructs (i.e. race, class, ethnicity) and these ‘… axes of power are intertwined as processes that construct and are constructed by the other’ ([28], p. 70).

Turning to gender in the context of leadership and management, [18] describe the uneven path of upward progression for women in organizations as a labyrinth, arising from the challenges associated with child care needs, racism, sexism, and discrimination on the basis of identity. A review of the literature indicates that the intersectionality of gender, with other dimensions of identity in the context of leadership, is under-researched in LMICs, particularly in Africa [5]. At the same time, a growing body of literature is emerging on modern forms of gender identity, particularly constructs of masculinity [11]. Social roles and constructs of masculinity, in particular in relation to domestic/homemaker and work/breadwinner, are increasingly challenged and redefined [56].

International discourse on social identities and their impact on leadership emphasise that a single social identity (e.g. gender) seldom operates in isolation, but intersects with other features [17, 51, 57]. Given the historical and health sector context of South Africa, we explored race and professional hierarchy alongside gender as influences on career trajectories, and on perspectives and experiences of leadership in a transforming health system.

We also considered the notion of transformational leadership [8], which speaks to a kind of leadership that encourages innovation and responsiveness, supporting a culture of learning and the promotion of value-based behaviour. This reflects the kind of leadership aspired towards nationally [46] and expressed in some South African provincial strategies [58]. In addition, we explored with managers how they enacted leadership in and through teams, reflecting the notion of distributed leadership [4] and in our analysis we drew on Le Deist and Winterton [35]‘s distinction between four intersecting leadership competency dimensions:

- cognitive - the ability to think critically and strategically;
- functional - the execution of specific technical aspects of leadership, such as relates to planning, service delivery, and human resource management;
- social - referring to understanding self and self in relation to others); and
- a higher-level synergistic, integrative competence which enables navigation of uncertainty and complexity in an ever-changing health system.

While these competencies speak to individual and team behaviours, equally importantly are the leadership-enabling health system capabilities that are crucial for nurturing an environment that allows individual leaders and the multiple teams that they form part of to thrive ([45], Baser and Morgan, 2005).

### Data collection methods and tools

Three approaches were applied within the in-depth individual interviews conducted: life history, vignettes and critical incident (CI) approaches. The life history approach allowed for in-depth exploration of participants’ career trajectories and leadership experiences [39]. The CI approach, on the other hand, enabled participants to self-report and reflect on significant events in their work which had influenced or been meaningful to their leadership experience [22, 32]. Vignettes were, finally, used to explore the sensitive issue of gender and/or race bias in leadership appointments and employment selection, as a way of probing more deliberately around our issues of interest. In order to allow enough time for participants to reflect on their backgrounds, practices and critical incidents, repeat interviews were conducted with 14 of the 19 participants. For the remaining five, and given their time constraints, we combined interview topics into just one interview.

### Data analysis

Transcriptions of interviews were uploaded onto Atlas t.i., a qualitative data analysis software. The analysis of the interview data drew on the conceptual ideas outlined above, following the four practical stages suggested by Marshall and Rossman [40] of ‘organizing the data’, ‘generating categories, themes, and patterns’, ‘testing any emergent hypotheses’, and ‘searching for alternative explanations’. The coding was guided by the principles of ‘comparative analysis’ [53], including the comparison of any coded element in terms of emergent categories and sub-categories leading to the identification of patterns. Each research team member provided input on the coding templates to ensure that the data were analysed appropriately. Transparency, validity, reliability, comparability, member checking, negative case analysis and reflexivity were used for building and ensuring methodological rigour [53]. Analysis occurred at the individual case level and across cases.

## Results

### Summary and relational mapping of study participants

Table 1 outlines the characteristics of the full sample. Of our 19 participants, 14 and five participants were interviewed in provinces one and two, respectively. Participants ranged from 40 to 60 years old, fourteen were women, thirteen black and twelve had a nursing professional background. By chance, the sample included participants who were closely related in a current or previous line-management capacity or had worked together in the same team. As participants spoke of their own leadership style and that of their teams, the inadvertent cross-referencing among some respondents provided some unexpected opportunities to triangulate experiences of each other during analysis.

**Table 1:** Participant summary

Participant number	Sex	Age	Race^a^	Position at the time of the study	Years in current position	Prof background	Number of years in management
1	Female	54	Black	Deputy Director	4	Professional Nurse	11
2	Female	40	White	Deputy Director	6	Professional Nurse	6
3	Female	57	White	Director	12	Professional Nurse	30
4	Male	41	White	Medical Manager of Hospital	8	Medical Doctor	8
5	Female	45	White	Deputy Director	4	Occupational Therapist	10
6	Female	61	White	Director	3	Medical Doctor	20
7	Male	60+	Black	Sub-Structure Director	5	Medical Doctor	20 years +
8	Female	49	Black	Director	7	Medical Doctor	10
9	Female	50+	Black	Sub-Structure Director	5	Professional Nurse	15 years +
10	Female	48	Black	Director	5.5	Registered Nurse	5.5
11	Male	62	Black	Director	17	Environmental Health Practitioner	30
12	Female	60	Black	Director	9	Professional Nurse	18
13	Female	56	Black	Director	3	Professional Nurse	16
14	Male	67	Black	Sub-District Manager	12	Medical Doctor	12
15	Female	48	Black	Deputy Director	1	Professional Nurse	11
16	Female	49	Black	Chief Director	2	Professional Nurse	11
17	Male	48	Black	Assistant Director	1	Professional Nurse	17
18	Female		Black	Director	10	Professional Nurse	20
19	Female	53	White	Deputy Director	3	Professional Nurse	17

^a^As noted earlier, the Employment Equity Act, uses racial classifications (i.e. white, African, Mixed-Race and Indian) to monitor transformation. However, for the purposes of this paper, we refer to participants as either black or white, since an analysis of participant experiences by more detailed race categories did not reveal marked differences

### Career trajectory into leadership

All 19 interviewees readily and enthusiastically recounted their career pathways, recollecting the factors and actors that played key roles at various points along their management journey. All had quite a chequered career pathway that took several twists and turns from their first entrance into management, to their current position. However, there was a distinctly different pattern discernible for doctors compared to nurses, emphasising the influence of professional hierarchy (see examples in Table 2).

**Table 2:** Management pathways of doctors and nurses

NARRATIVE 1 (P16: female, black, nurse)	
At the age of 18, she started as an enrolled nurse and progressed to general nursing and midwifery and later studied advanced midwifery. Her first management post was as a nurse-in-charge of a maternity ward, but she left the position on account of her husband who received a job promotion, which required relocation. During that time, for approximately 3 years, she worked in non-management positions in clinics and hospital and tutored at a nursing college). She then got a position in government as sectional manager responsible for all municipal clinics and remained there for two-and-a-half years. She continued to study further (management, financial management). With restructuring of the provincial Department of Health, she applied for the post of sub-district manager and remained in the post for nine months before applying for and getting the post of chief executive officer (CEO) of a hospital and remained in post for 3 years. She then applied for provincial post of director of PHC and remained for a further 3 years. She resigned again, on account of her husband who was relocated again and worked as director of Primary Health Care (PHC) in an urban district and later became an acting chief-director (very senior management position). She finally applied, successfully, for a chief director post in 2014.	
NARRATIVE 2 (P14: male, black, doctor)	
After qualifying as a medical doctor, he worked as a private general-practitioner (GP). He was also involved in political activism, was a counsellor in the local municipality and served on the health committee. As a GP, he was “involved in running the hospital” and later stepped into a formal management position as CEO in managing the hospital. He still maintained a private practice and managed the hospital part-time. After almost a decade, he relinquished his private practice to manage the hospital full-time. Since 2003, he is also a sub-district manager overseeing 8 primary care facilities.	

All doctors in our sample went directly from being a clinician to a hospital manager (equivalent to a level 2 or 3 manager), which placed them at a middle management level from the outset of their management careers. They all followed a steady upward trajectory and mostly went on to fill senior management positions. In contrast, nurses mainly landed their first management job as an assistant director or a primary level facility manager (equivalent to a level one manager). They had to climb the ladder from the lowest level of management and their journeys took many more twists and turns over several years, often entailing more lateral moves than their doctor-colleagues. The majority of nurses in the sample were at the level of deputy director (the second lowest level of health manager in the South African health system), as opposed to that of a director (regarded as a senior management position), a position held by most of the doctors in the sample. These different career patterns suggest the intersection of professional hierarchy with gender as an influence over their experiences. That most nurses are women also illustrates the inextricable link between the gendered nature of the health workforce and the way in which gender and professional hierarchy intersect at the level of leadership.

Several women spoke about being the first to enter a particular position. As the interviews took place just after two decades of democracy, women, and black women in particular, were, for the first time, occupying positions that were previously reserved for Whites, males or doctors. Some examples are:

*‘[I was the] first mixed race professional nurse in one of the hospitals (P13, female, black, nurse)’.*

*‘[She was] one of the first women and nurses to occupy a senior management position in her organisation’ (P3, female, white, nurse).*

*‘[I was the] first black nurse to become a school health nurse and also the first black nurse to launch and head up a district office (P18, female, black, nurse).*

As these quotes illustrate, in addition to gender and professional hierarchy, race also played an important role in the career trajectories of women managers. These women were pioneers in their organisations and did not have other women managers with the requisite experience to turn to for advice and mentorship. One of these pioneering women managers has since become a role-model and mentor to many, as reported by several of our interviewees.

Similar to the career-pathway differences referred to in Table 2 for nurses as compared to doctors, black women managers faced more obstacles and took longer to get into management in comparison to their white counterparts, facing a ‘triple-challenge’ of gender, professional hierarchy and race in their career trajectories. With the exception of one black woman manager, gender played a role in the difficulties they faced in their careers. For the single exception, she attributed strong family support and positive affirmation by her father from a young age as the reason gender was not an obstacle in her professional experiences and trajectory. In another exceptional circumstance, one female manager cited her race as a negative influence. She explained that, ‘in the new democratic South Africa [she is..] unlikely to progress beyond her current mid-level position’ (P19, female, white, nurse). In this study site, white managers were in the minority.

Gender also played an important role in the personal career choices women managers made, regardless of race. Similar to her counterpart in Table 2, a white female manager indicated that she and her husband both had aspirations of becoming surgeons, but when they had children, she decided to put her career on the backburner to allow her husband to follow his dreams. She opted for a career in management instead, as she perceived the hours to be more family-friendly.

The five males in the sample experienced an interesting mix of negative and positive influences from the social identities we explored. A black male manager in Province 2 spoke of having experienced many societal, professional and now managerial prejudices as a ‘minority’ person based on his professional identity and gender. He spoke strongly to how, as a male nurse, he endured prejudices from female colleagues and he also had to cope with similar prejudice about his chosen occupation in his family and social circles. In his management position he was the only male manager amongst older female managers:

Males in this are so submissive to women…They are so submissive in such a way that they ended up losing it and deciding “I’m going just to be quiet” so they are not being felt. so, me having to come here and telling them [women] “..No, you are wrong there, let’s do it this way or come sit, let’s plan.” So it becomes a challenge for me because they are not used to a male telling them “No, no, no”. (P17, male, black, nurse);

Three other black male managers highlighted race as having played a role in their career opportunities and choices. During apartheid, one left to pursue a career in a neighbouring country due to racial prejudice in South Africa and later returned post-democracy; another spoke implicitly of not being part of the ‘in-crowd’ of males in his department as a result of racial difference; and a third, whilst he did not mention issues of race, recounted how his strong anti-apartheid and activist roles in health matters in the pre-democracy period automatically made him the first choice to run a local hospital post-democracy. The fifth male, white and a doctor, did not allude to any difficulty in his career trajectory as a result of these social identities.

### Practices of leadership

Participants’ practices of leadership, which were a mix of positive and negative experiences, were explored through the critical incidents. Participants described a diverse set of critical incidents, ranging from challenges within their own sphere of influence (e.g. team work and organizational culture, management styles) to events which they had no or limited influence over, but were experienced as a crisis (e.g. unexpected increase in patient load at a hospital, sudden resignation of a staff members) and in some instances as a catastrophe (e.g. death of colleagues).

Despite the variation in incidents, managers all described their impact both on personal leadership practices and wider team performance. In the following quote, the participant (district manager) was concerned both about the poor performance and a reputational crisis of her district when the manager for Human Immuno-deficiency Virus (HIV) and Acquired Immuno-deficiency Syndrome (AIDS)/Sexually Transmitted Diseases (STDs) /and Tuberculosis, known as the HAST manager resigned. Her strategy for addressing the problem was to systematically address and intensify all the components of the HAST programme and work with multiple actors to achieve this:

“*It was like [Name of district]…is taking the whole province down….it means I’m not doing my job, you know, because the buck stops with me. I’m the one taking accountability, so I cannot afford to be disgraced in, in a provincial meeting that’s a no-no for me… Then we had to work very hard; go do campaigns…you have to intensify the finding out…how you are going to get patients…the diagnostic part has got to be intensified..once that is pushed then the ones on antiretroviral therapy (ART) will also be improved, ones that are on ART are there we also do adherence clubs…what we needed to do also was to also increase the community health care workers, the volunteers; because they are the ones that go to the houses…to intensify our relationship with the partners.”* [P16, female, black nurse].

The above critical incident illustrates day-to-day management issues that are potentially predictable, but which some managers nonetheless experience as crises. The example highlights how reputation, performance and accountability drive leadership practices. In contrast, in unanticipated crises such as the death of a colleague, health managers typically do not have contingency plans. Responses to such events may demonstrate strong team reactions (such as teams going beyond their scope of work and even outside of their usual geographical boundaries to support one another), but also test a leader’s decision-making and strategic-thinking abilities.

“ .. So it’s a huge gap now the clinics must go on so– one day clinic S will go and help; two days Clinic V will come and help. So within a week they organise a whole group who can help; also with their little capacity, but they go and help”. [P3, female, White, nurse]

This critical incident referred to above, relates to the deaths of several colleagues in one accident. It reveals three important facets of leadership in the time of crisis. First, crises are opportunities for strengthening relationships and networks of support. Second, the enactment of distributed leadership. Third, the notion of ‘meta-competence’, as illustrated by the continued functioning of the clinics despite the extreme crisis brought about by the tragic loss of several staff members.

Through and beyond the critical incidents, we see examples of managers with different leadership practices which have important implications for themselves and their teams. For example, one district was described to have a ‘pervasive toxicity’ which was attributed in large measure to the leadership practices of the district manager. This toxicity was, however, significantly reduced by a supportive coaching intervention for the leader (Table 3). The example in Table 3 illustrates how leadership difficulties, such as were experienced by the manager, can provide opportunities for personal growth and development through self-reflection and a willingness to change with ripple-effects for transformation at the organizational level.

**Table 3:** Toxic leadership practices: an opportunity for transformation at the personal and organizational level

This leader was subject to significant challenges as a first-time hospital manager and reported experiencing significant prejudice from white male doctors, who questioned her appointment as a black woman. Some of these difficulties continued into her current role, demonstrating the negative reinforcement of inadequate support and capacity to take on leadership roles, especially when compounded by prejudice and discrimination. Her willingness to open herself and her team to a coaching intervention catalyzed positive change in her leadership practices and the organizational culture. Prior to the coaching intervention, interactions with the manager were described by a colleague as ‘*going into hell’.* Following the intervention, the same colleague described interactions with her as *‘enjoyable*’ and where he ‘*looked forward to going to her office’*.	

In one province, several managers at different levels spoke of being supported and mentored over many years by their managers. They spoke fondly of those who ‘gave them a chance’, mentored them throughout their professional and managerial career, and who remain close colleagues and role models to date. Despite movement into different teams and different districts, or between levels of management, these relationships endured and resulted in an informal ‘network of managers’ who value and support one another. This kind of support seemed to be independent of any of the social identities that we explored.

### Perceptions of ideal leadership and leadership practices

Perceptions influence behaviours and we further explored with managers their understanding of leadership and what they regarded as important or ‘ideal’ leadership characteristics. Interestingly, all interviewees understood leadership and management as a continuum and that, as managers, they had to execute a set of daily practices where some fit more in the domain of leadership and others more under management. Some weighted the transactional aspects of their responsibilities strongly, such as having to set guidelines and follow processes and execute the administrative requirements of their portfolios whilst others spoke more deliberately of the transformational aspects, such as working towards serving the public, setting strategy and being visionary.

A male manager eloquently described his perceptions of his multiple roles in one day, where it was clear he draws on different competencies (cognitive, technical and social) as he changes tasks, whilst trying to hold it all together to create stability amidst multiple demands (meta competence). He demonstrates the ‘every day resilience’ of frontline managers [24] and the inextricably-linked ‘leading’ and ‘managing’ daily activities of a manager (Mintzberg, 2005). He also speaks of his practices, shaped by his underlying personal values and specifically those which embrace a balanced family-work life, a pursuit which is stereotypically associated with women managers:

“After your interview today there will be an human resource (HR) person coming to me; and then the Finance Head, and then I have an unhappy staff member because of work relationships in the workshop. Then I have a document to submit as a complaint for the Minister by 8 o’clock tomorrow morning; that is a continuous thing… where the HR person thinks HR all the time; while I’m writing an email on HR, I will answer a telephone call on information management, and my finance person. You have to change all the time continuously; and if you don’t have a structure in place – a system in place on how to deal with the various streams of work in your area – under your control - it is going to travel around in your head all the time; and you continuously think” “What have I missed, what should I…what should I have done today. What deadlines are there today; tomorrow, or in a week?” Uhm…yeah it’s – one good thing I do is I leave at 16:00; 07:30 until 16:00 I work as good as I can. But I leave here at 16:00. I go and pick up my kids and I am dad; and I preach it to my staff all the time. [P4, male, white, doctor]

**Fig. 1:**
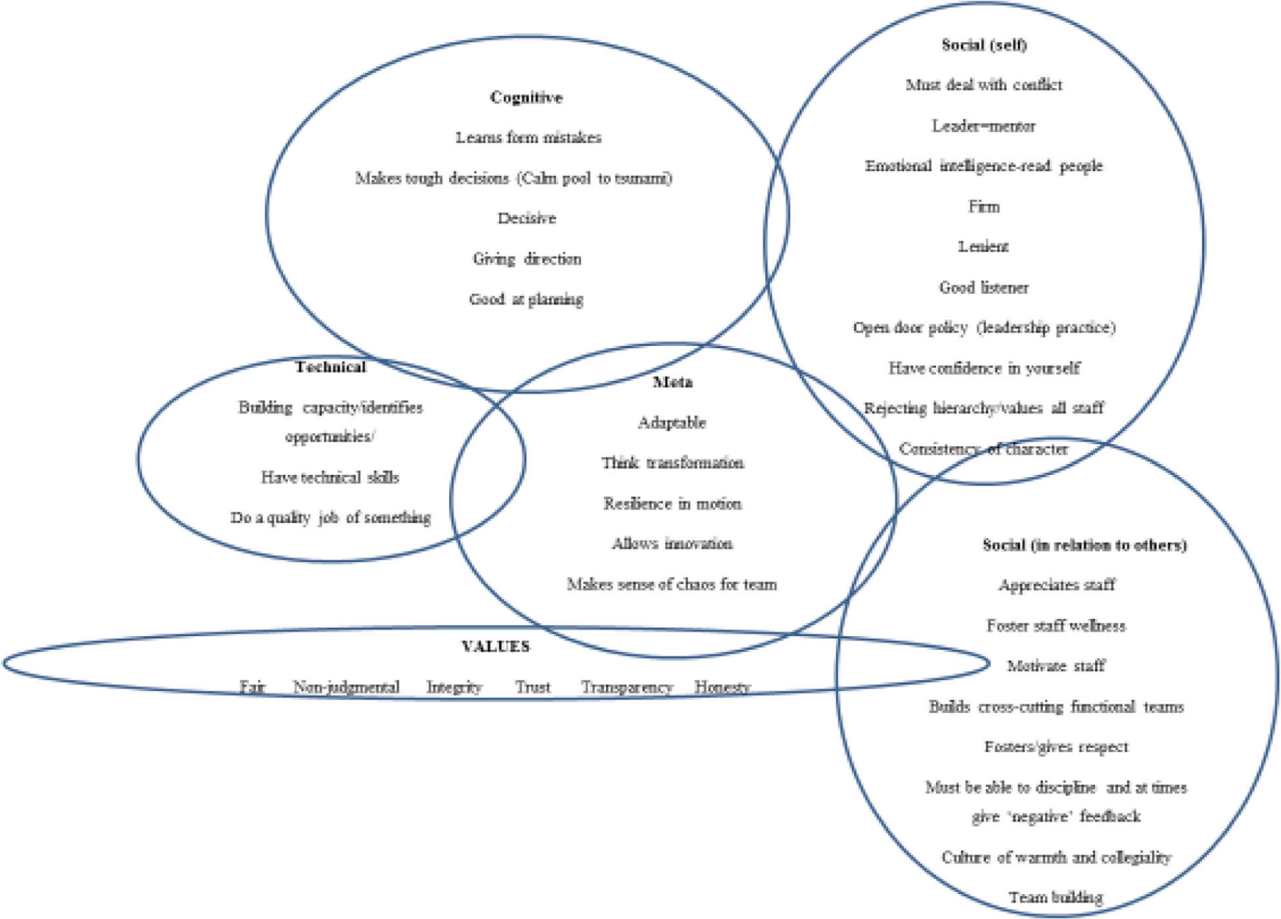
Manager’s perspectives on good leadership characteristics

We analysed the words and phrases used by managers to describe good leadership characteristics (behaviours) and placed these into the four Le Deist and Winterton competency domains (Fig. 1). Most leadership behaviours identified by managers fell under social competencies, illustrating their strongly relational notion of leadership. The commonly accepted notion that leaders require high emotional intelligence also permeated the perspectives of the managers in this study. Managers also expressed a number of values that they followed including ‘..fairness, integrity, trust, transparency, honesty and being non-judgmental..’. For some participants, these values were reportedly derived from particular family members and/or their religious beliefs. In one study site, managers explicitly referred to values contained in their organisational behaviour charter, suggesting that converging personal and organisational values may have a reinforcing effect.

Managers also spoke of ‘ways of leading’ that suggested a preference for distributed leadership within their teams and in the organisation more broadly, as indicated by phrases such as ‘…rejecting hierarchy; valuing all staff; leadership and followership; leading from behind; supporting discretionary power; and being inclusive’.

All participants recognised the importance of teams and team leadership and the importance of ensuring that teams, as a collective, possess the necessary leadership traits**.** In keeping with their implied notions of distributed leadership, they often spoke about their willingness to delegate and allowing others to get on with the job, and also explicitly named others as leaders in their own right. In the critical incidents, they identified practical examples of how they acknowledged and encouraged others in their teams to take leadership. Death and illness were the predominant ‘critical incidents’ mentioned and in these extreme and tragic circumstances (two managers in particular experienced multiple unexpected deaths due to accidents, within their teams), they spoke passionately about how teams drew together, relationships were strengthened, and team resilience increased. Two others spoke about the illness of their senior managers, one of whom subsequently died, and how that impacted on them. One was even inspired by her late manager to take up the leadership position that he vacated.

Managers largely spoke of the leadership and team characteristics in a ‘gender-neutral’ way and also did not raise race or professional hierarchy as important influencers. Only four managers volunteered gender as a factor in influencing the kind of values that managers’ hold and aspire towards. One senior male manager reported that women managers were more caring and nurturing and had more effective ways of communicating and managing difficult one-on-one relationships in the workplace. A female manager posited that:

.“… women are very tough; but they are also empathetic. …..When I looked at all women that are being put in power most of them, they take organisations very far. It’s not because I’m a woman; but that’s my view. When you look at organisations that actually crumble, in most cases they crumble in the hands of males, most of them”. [P16, female, black, nurse]

With reference to their leadership positions, women managers had mixed perspectives as to whether their gender had negatively influenced their ability to advance in their careers. One participant spoke very forcefully about being disadvantaged by the presence of a ‘boys club’ in one study site, whilst others spoke favourably of how men in management positions had supported and helped advance their careers.

Managers had perspectives of what constituted ‘ideal’ characteristics and practices in a leader, usually recounting these in managers they admired and respected. The characteristics that emerged were more pragmatic and functional than their aspirational notions of what leadership is. A frequently admired characteristic was being ‘decisive’ regardless of the nature of the decision. One manager when she assumed an acting senior management role was advised by her boss, “.*.whatever you do, please just make a decision even if it’s the wrong one. I will back you up”* (P10, female, black, nurse). The next most common trait that featured in almost every interview was that of being a role model. They described how ‘being exemplary’ and ‘walking the talk’ as very important. Most participants had a specific role model in mind and it was usually someone more senior, who had mentored or supported them at different points during their career. Further ideal characteristics mentioned by managers included: ‘Being firm, technically good, accountable, know[ing] the policies and procedures, and being balanced’. One manager referred to herself as: “*being a calm stream and Tsunami at the same time*” (P9, female, black, nurse), suggesting the varied behaviours that leaders have to exhibit.

An analysis by gender, race and professional hierarchy did not reveal any discernable patterns: we did not find any differences in how men and women, doctors and nurses and black and white participants spoke of ideal attributes.

## Discussion

In this paper we sought first to explore the influence of gender on career trajectories into formal managerial positions and second to explore whether gender influences practices and perceptions of leadership. Through in-depth qualitative analyses, we confirmed the intersecting influences of social identities as espoused by other authors and found that gender did not act alone in shaping pathways into management, but intersected with, and in some instances was over-shadowed by, race and professional hierarchy. We found that the strongest influence of these social identities emerged with respect to career pathways. The inter-twining of the repressive race-based history, the persistently patriarchical society and the medical hierarchy strongly shpaed career opportunities and pathways.

Professional hierarchy was the most significant influence on the level of ease participants experienced in progressing towards management, with far more challenging experiences reported by nurses and especially black nurses. The broader human resource and management and leadership literature is fairly silent on this constraint over managerial careers. Further research in South Africa and other settings would deepen our understanding of this phenomenon.

The influence of gender on career trajectories, and in particular gendered roles within dual-career households, was observed in our study, with some female professionals prioritising their male partners’ careers ahead of their own. This reflects wider experience of women in healthcare [26, 41]. This prioritisation of male partners’ careers was also observed in professional clinical women who sometimes chose management over clinical jobs to cope with family demands; a commonly reported experience of professional women in health care [52, 54]. At least one of the male respondent challenges convention, in keeping with the emerging literature on masculinity [56], where he strongly advocated for a work-life balance and the prioritisation of child care and family time.

More unique to the South African experiences reported here was the strong confluence of race and gender in influencing career pathways. Black participants, both men and women, described the role of pre-democracy apartheid policies in limiting their higher education and vocational options, and contributing to complex and protracted higher education and career pathways into, finally, management. As Bunting describes, “Under apartheid, higher education in South Africa was skewed in ways designed to entrench the power and privilege of the ruling white minority” [7]. black women (nurses), moreover, faced the triple challenge of profession, gender and race, in navigating a career into and through management. Our respondents recounted many examples of the triple discrimination that they had to face, and how, enabled by progressive employment equity policies in the post-apartheid period, they were often the “first” black leaders occupying positions previously reserved for white males, often doctors. The supportive role that families played in enabling female black managers to pursue their education and careers was frequently highlighted. In the process, they were able to move away from their traditional gender roles as primary care-givers and mothers. Employment equity and supportive home environments, at least for this cohort of women, countered the leadership “labyrinth”^3^ of gender-based barriers hampering women’s upward mobility in management [41].

We also had a few examples where experiences and practices did not follow the expected pathways such as the black male nurse manager that experienced discrimination in a black female-dominated management environment, the male manager who strongly advocated for family-work balance and the female senior manager for whom gender was a non-issue. In addition to the recurring influence of gender, was the consistent finding that gender almost always intersected with the social identities of race and professional hierarchy in influencing experience.

Our study shows that South African managers hold contemporary notions about the nature and form of leadership, as expressed in the more recent leadership literature. Almost without exception they spoke of leadership as extending beyond the domain of individuals to teams, in contrast to notions of *the* individual leader that still dominates the management and leadership literature. The importance of teams and teamwork manifested both in concept and in practice as shown in the critical incidents [6, 37, 38]. Respondents also implicitly referred to distributed leadership in teams and alluded to the importance of recognising leadership in others, helping others in their leadership careers and valuing the recognition of their leadership by those senior to themselves. Finally. managers emphasised the impact of systemic factors on their leadership and spoke about the challenging effect of multiple and complex demands, resource constraints and uncertainty that they have to deal with daily [24].

In exploring their perspectives on ideal and desirable leadership characteristics, the matrix of domains espoused by LeDeist and Winterton, were repeatedly articulated [35]. Reflecting the understanding that leadership is a social construct and process, respondents identified social competencies, those of understanding themselves and how they relate to others, as the strongest and most favoured set of competences they felt leaders should have. Strong social competencies also support a more transformational approach to leadership, which contrasts with the transactional approach common in large bureaucracies. Transformational leadership requires managers to have an awareness of the importance of the organisational or system ‘software’ and in particular the intangible software, which includes elements such as trust, respect, integrity, to which many managers alluded [14, 20, 29]. Although not strongly articulated in the findings of this research, gender may contribute to recognition of the importance of social competency: some women respondents, and at least one of the male managers, explicitly suggested that women managers favour communication, caring and nurturing –typifying the gendered behavioural norms associated with women [9, 31].

An unexpected and particularly interesting finding was the sense of a network of supportive managers given leadership longevity and stability in one of the provincial sites in particular. Some of the participants in the interview cohort were regarded as role models by other participants. For one manager, role modelling was also a personal aspiration. This illustrates the potentially powerful effect of having cohorts of managers that embrace similar values, aspirations and behaviours, as these eventually become the organisational culture of ‘how we do things around here’. This ‘inter-generational’ role-modelling and mentoring as well as peer-support, may have the potential to grow a community of leaders adopting particular leadership practices, which in turn may promote an organisational culture that itself nurtures such leadership [19, 36]. Furthermore, organisational processes that recognise differing social locations, actively mitigate against structural barriers due to social identities and promote support and opportunities are essential in the building of transformative work environments. Does gender influence this? Our study hints at this influence, but in order to grow stable, resilient, cohorts of leaders across health systems, it is more important to recognise and support talent and potential, irrespective of gender, race or profession.

In terms of leadership and leadership development, the findings of this study point to the need for further consideration of the following issues and processes:

- The need for job-design to be sensitive to and incorporate issues of gender, race and professional hierarchy, in order to support candidates’ readiness/preparedness for positions of leadership in healthcare organisations;
- The design and content of healthcare leadership development programmes to explicitly consider and address issues of gender
- Support systems (i.e. mentoring, coaching) for all healthcare leaders, especially early-career in the presence of gender, race and profession-based bias
- The importance of managing diversity in healthcare organisation.

## Conclusions

Gender, in intersection with race and professional hierarchy, is one of many influencing factors over the mix and composition of management cadres in the public health sector of South Africa. Given the small size of this exploratory study, a number of aspects cannot be conclusively and empirically confirmed. However, the insights that emerged from this study help us understand a little better the complex dynamics of leadership and the intersection of three social phenomena (gender, race and professional hierarchy) in the selected settings. Their continuing influence in South African and other societies, albeit in different ways, suggests the value of further investigation of how they shape health leadership and finding ways to mitigate their potentially negative impacts.

## Abbreviations

ART: Antiretroviral therapy
CEO: Chief executive officer
CI: Critical incident
GP: General practitioner
HAST: Human Immunodeficiency Virus (HIV) and Acquired Immune-Deficiency Syndrome (AIDS), Sexually Transmitted Infections (STI) and Tuberculosis
HCT: Haematocrit
HR: Human resource
LMICs: low- and middle-income countries
PHC: Primary Health Care
SDG: Sustainable Development Goals

## Footnotes

1 An important disclaimer, which echoes the seminal historical paper of Coovadia et al. [12] referenced here, is that the terms used for the different races are consistent with those in common use and employed by the national census and do not imply acceptance of racial attributes of any kind on the part of the authors*.* In the Employment Equity Act, Black refers to citizens of ‘African, Mixed-Race and Indian’ origin and the persistent use of these racial classifications was justified as needing to monitor the transformation in the employment practices across the country, in order to redress the previous racial imbalances on employment and opportunity.

2 Senior health managers as distinct from level 1 and 2 managers whose key roles relate to managing teams and other managers and top level managers who might head departments of health [23].

3 Eagly and Karau refer to the ‘labyrinth’ as the complex and varied set of challenges that women face on their journey to leadership (11)
